# Durable Response to Atezolizumab in Extensive-Stage Small-Cell Lung Cancer Leading to 60 Months Overall Survival: A Case Report

**DOI:** 10.3390/curroncol31070271

**Published:** 2024-06-27

**Authors:** Freeman Paczkowski, Jacques Raphael, Claire Browne

**Affiliations:** 1 Schulich School of Medicine & Dentistry, Western University, London, ON N6A 3K7, Canada; fpaczkow@uwo.ca; 2 Division of Medical Oncology, Department of Oncology, London Health Sciences Centre, Western University, London, ON N6A 5W9, Canada; claire.browne@lhsc.on.ca

**Keywords:** small-cell lung cancer, extensive-stage small-cell lung cancer, durable response, atezolizumab, long-term survival

## Abstract

Small-cell lung cancer (SCLC) remains a disease with poor prognosis, particularly in extensive-stage SCLC (ES-SCLC). Current standard-of-care treatment includes chemotherapy with platinum agents and etoposide plus immunotherapy with atezolizumab or durvalumab, which has achieved a mean overall survival of 12–13 months in clinical trials. However, long-term survival in ES-SCLC, even with the addition of immunotherapy, continues to be rare. We present the case of a middle-aged male patient diagnosed with ES-SCLC who was treated with four cycles of induction chemotherapy (carboplatin and etoposide) and atezolizumab, starting maintenance atezolizumab every 21 days thereafter, and thoracic radiotherapy. After 9 months, he experienced mild disease progression and was rechallenged with six cycles of carboplatin and etoposide with continued atezolizumab. Subsequent imaging showed near-complete disease resolution which has been sustained since. He has continued on maintenance atezolizumab since diagnosis and has achieved 60 months overall survival and 44 months progression-free survival. Throughout treatment, he has maintained a high functional capacity and only experienced one immune-related adverse event. Our patient is representative of a small subset who are capable of achieving durable responses to immunotherapy and his case highlights the need for further research to elucidate the clinical and biological factors driving this response.

## 1. Introduction

In 2020, lung cancer was the leading cause of cancer death worldwide, representing 18% of deaths overall [1]. The two main types of lung cancer are differentiated based on pathologic characteristics: non-small-cell lung cancer (NSCLC) and small-cell lung cancer (SCLC). SCLC is less common, representing approximately 15% of lung cancer cases; however, it is typically more advanced at presentation and carries a worse prognosis [2]. Staging for SCLC is divided into limited stage (LS-SCLC), when the extent of disease is confined to one radiation field, and extensive stage (ES-SCLC), where a radiation field is exceeded [2]. The median overall survival (OS) for LC-SCLC and ES-SCLC is approximately 17 months and 12 months, respectively [2].

First-line therapy for ES-SCLC has historically included a platinum-based chemotherapy regimen, consisting of four-to-six cycles of cisplatin or carboplatin plus etoposide in North America [2]. Recently, treatment for ES-SCLC expanded with FDA approval of new immune checkpoint inhibitor (ICI) atezolizumab in 2019. Atezolizumab is a monoclonal antibody against programed cell death ligand-1 (PD-L1), which can be highly expressed on the surface of tumor cells and utilized to evade immune-mediated destruction [3]. In IMpower133 (2019), atezolizumab with standard chemotherapy versus placebo and standard chemotherapy achieved 12.3 vs. 10.3 months median OS with a hazard ratio of 0.77 (95% confidence interval 0.54–0.91) and 51.7% vs. 38.2% one-year survival [3]. These results, along with the results from CASPIAN (2020), which showed similar survival benefits for the ICI durvalumab, led to the establishment of chemoimmunotherapy as the gold-standard treatment for SCLC [4,5]. However, despite the modest survival benefits offered by ICIs, outcomes generally remain poor for SCLC patients [5].

Here, we report a case of an adult male patient with ES-SCLC who has achieved a durable response to atezolizumab employed as part of induction and maintenance therapy. This patient is still alive at the time of this report and has maintained a high quality of life since diagnosis.

## 2. Case Presentation

**Figure 1:**
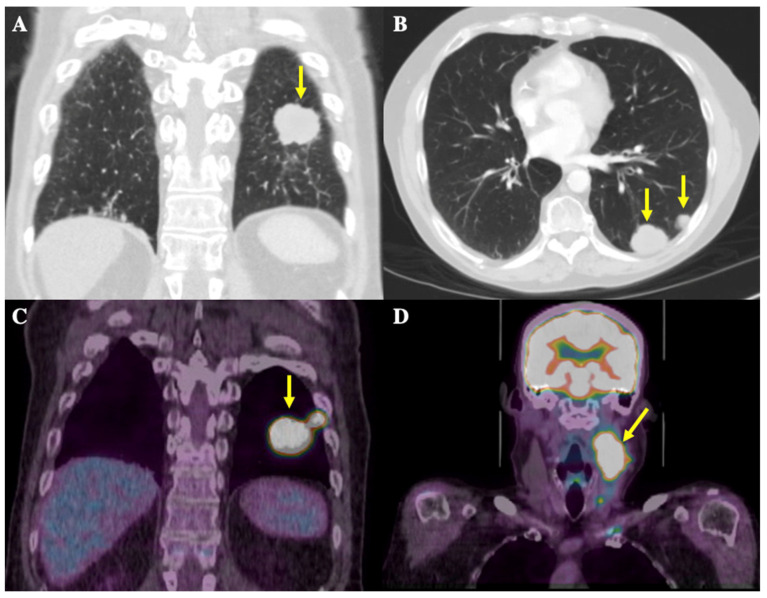
CT thorax and 18-Fluorodeoxyglucose (FDG)-PET scans at initial diagnosis. Yellow arrows depict radiological findings. (**A**) Coronal image from the CT thorax scan showing the largest mass in the left posterior lung base, measuring 2.8 × 3.4 cm in size. (**B**) Axial image from the CT thorax scan showing an additional view of the metastatic nodules in the posterior left lung. (**C**) Coronal images of the thorax on the FDG-PET scan show the largest FDG-avid lung mass, in the left posterior lung, and the adjacent hypermetabolic nodule. (**D**) Coronal images of the head and neck on the FDG-PET scan show a left-sided nodal conglomerate in the neck measuring 3.4 × 4.3 × 6.5 cm along with adjacent hypermetabolic lymph nodes measuring 0.5–0.9 cm (not well demarcated in image).

In the summer of 2019, a 58-year-old male patient presented to a walk-in clinic in London, Ontario, Canada for investigation of a 1.5 cm-by-2.5 cm, painless, left-sided neck mass. A CT thorax scan in September 2019 showed a large mass located posteriorly at the left lung base measuring 2.8 × 3.4 cm in size along with multiple other smaller pulmonary nodules ranging from 5 to 15 mm, present in the left base, left upper lobe, right middle lobe, and right hilar and pre-carinal regions, highly suspicious for metastatic disease (Figure 1A,B). The pathology obtained via fine needle aspiration (FNA) of the neck mass was consistent with metastatic SCLC. Staging workup included a magnetic resonance imaging (MRI) head scan, which showed no intracranial metastases, and a positron emission topography (PET) scan. This scan showed increased uptake in the largest lung mass as well as multiple right and left-sided pulmonary nodules ranging from 0.7 to 1.7 cm in size, and in the neck mass plus multiple left-sided cervical lymph nodes ranging from 0.5 to 0.9 cm in size (Figure 1C,D). These results confirmed the diagnosis of ES-SCLC.

Given the patient’s diagnosis of ES-SCLC, palliative-intent treatment with first-line combination induction therapy of atezolizumab with carboplatin and etoposide was initiated. The induction treatment for this patient consisted of four cycles of carboplatin (681 mg IV, day 1), etoposide (350 mg PO daily, day 1–3), and atezolizumab (1200 mg IV, day 1) every 21 days from Nov 2019 to Jan 2020. The complications encountered during induction treatment included one admission for febrile neutropenia after cycle three of chemotherapy. This event prompted a 25% reduction in carboplatin (563 mg IV, day 1) and etoposide (200 mg PO, day 1–3) doses in the final cycle of this phase. Other than the hospital admission, this initial regimen was tolerated well, as the patient experienced no immune-related adverse events (irAEs) or functional decline. He actually noted functional improvement due to shrinkage of the neck mass, and felt well throughout induction treatment. He was then transitioned to maintenance atezolizumab every 21 days (1200 mg IV).

**Figure 2:**
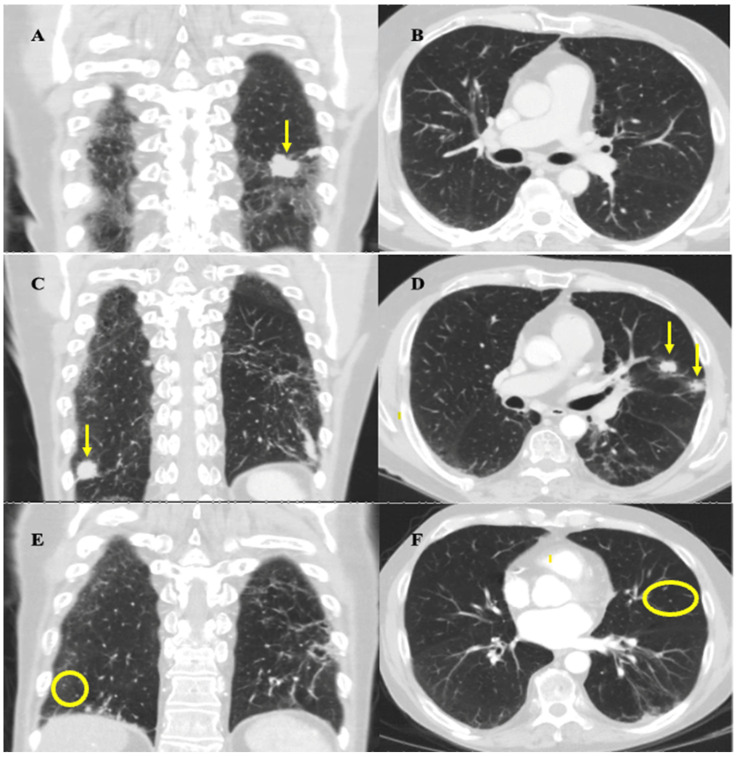
Coronal and axial mid-lung CT thorax images highlighting important stages of disease progression and response to therapy. Images were obtained at approximately the same slices for each time point. (**A**,**B**) February 2020 imaging after induction chemotherapy and radiation showing treatment response. (**A**) Arrow highlights reduction in size of largest left lung nodule from 3.4 × 2.8 cm to 2.7 cm × 1.6 cm. (**B**) No evidence of metastatic nodules in mid-lung zones of left and right lung zones at the level of the hilum. (**C**,**D**) September 2020 imaging showing progression of disease. (**C**) Coronal view shows resolution of largest left lung nodule but appearance of a new right lung nodule near the costophrenic angle, indicated by the arrow, measuring 1.9 cm. (**D**) Axial view shows appearance of two new metastatic nodules in left upper lobe, indicated by the arrows, each measuring approximately 1.4–1.5 cm in size. (**E**,**F**) March 2021 imaging post rechallenge with chemotherapy and atezolizumab showing near-complete resolution of disease. There is resolution of the nodules seen on the September 2020 scan (circles highlight locations of previously seen, now resolved, lesions). Residual scarring was also observed.

The follow-up CT thorax scan on February 2020 showed a partial response to this initial regimen, as most of the metastatic pulmonary nodules decreased in size, including the largest nodule, which decreased from 3.4 × 2.8 cm to 2.7 cm × 1.6 cm, and there was no evidence of new metastatic disease (Figure 2A,B). After a multidisciplinary case conference discussion verifying safety, the patient underwent radiation therapy to the left lower lobe pleural-based lesion, left-sided hilar and mediastinal lymph nodes, and left neck from March to April 2020 (30 Gy in 10 fractions to each site). This decision was made with the intent to prolong survival and was informed based on the results of the CREST trial [6]. Maintenance atezolizumab was held during radiotherapy to decrease the risk of compounding side effects. Radiation was well tolerated overall, with his main side effect being mild odynophagia which did not impact nutrition. The patient declined to undergo prophylactic cranial irradiation (PCI) and opted for an MRI surveillance approach instead.

In July 2020, standard CT surveillance showed a mixed response to treatment. A CT thorax/abdomen scan showed a decrease in size of the largest nodule at the left lung base (now 1.3 × 2.0 cm) and right hilar and subcarinal lymph nodes, but an increase in size of the pre-existing nodules, along with the appearance of new suspicious nodules. The decision from our institution’s thoracic tumor board meeting in August 2020 concluded that these findings were more in keeping with true progression as opposed to pseudoprogression. In Sept 2020, a CT thorax/abdomen scan demonstrated further disease progression by highlighting the size increase of the metastatic nodules uncovered in the July 2020 scan (Figure 2C,D). The patient developed mild shortness of breath, but otherwise felt well, and functional status remained unchanged. This progression prompted the patient to undergo rechallenge with induction chemotherapy along with continued immunotherapy. The decision to rechallenge with platinum chemotherapy was made as he experienced a platinum-free interval of greater than 6 months (approximately 9 months) before relapse, and was therefore platinum chemo-sensitive [7]. Atezolizumab was continued because, given the mixed response, it could not be concluded that this medication had definitively failed. Given these factors, and his high tolerability for atezolizumab up to this point, rechallenge with the induction chemotherapy regimen and subsequent continued immunotherapy was deemed the best approach for this patient.

He completed six cycles of carboplatin (225 mg IV, day 1), etoposide (143 mg IV, day 1–3), and atezolizumab (1200 mg IV, day 1) every 21 days from Oct 2020 to Jan 2021, with the duration chosen to maximize the effectiveness of the rechallenge and given his ongoing tolerance. Chemotherapy was initially dose-reduced by 25% as in his last cycle of the first round, and a further dose reduction to 35% of carboplatin and etoposide on cycle three was required due to the development of anemia (carboplatin 195 mg IV, etoposide 124 mg IV). He experienced mild fatigue throughout the rechallenge, and additional symptoms of dysgeusia and loss of appetite at the last cycle of treatment. A CT head/neck scan performed in December 2020 showed resolution of the left-sided neck mass and associated lymphadenopathy. A CT thorax/abdomen scan performed in March 2021 demonstrated complete resolution or reduction in size of most metastatic pulmonary nodules, and the resolution of mediastinal lymphadenopathy (Figure 2E,F). These results demonstrated a positive response to rechallenge. Maintenance atezolizumab was subsequently restarted with the plan to continue surveillance imaging every 3–4 months.

To date, this patient has received 72 cycles of atezolizumab since diagnosis as part of induction, rechallenge, and continued maintenance therapy. He has tolerated this excellently, with mild intermittent fatigue and changes in appetite as his only recurring symptoms. There has been no evidence of further disease progression documented on any follow-up CT thorax/abdomen and neck scans since completion of the second round of induction chemotherapy. This patient has never developed metastasis to the brain, as evidenced by negative MRI head surveillance and an absence of neurologic findings. MR head surveillance was initially planned to occur every 3 months for 1 year; however, the then-ongoing COVID-19 pandemic caused significant issues obtaining imaging after 6 months of surveillance. Imaging was continued as frequently as possible until Jan 2022, at which point he remained clinically well; due to a lack of guidelines in this space, it was decided after discussion that further cranial imaging would be symptom-guided. He remains free of neurologic symptoms.

Other than one previous admission for febrile neutropenia, the only major medical complication this patient has experienced throughout treatment has been medication-related osteonecrosis of the jaw (MRONJ), present at the right and left mandible and left maxilla. This was precipitated by long-time IV Zoledronate use, which was prescribed in February 2020 for hypercalcemia. This medication was discontinued after its 15th monthly cycle in Oct 2021. He underwent marginal resection of the affected areas in Feb 2022 and repeat resection of the left mandible in Dec 2022. Additionally, the patient’s atezolizumab was held in June 2023 (after cycle 56) due to development of transaminitis, with grade 3 elevation in GGT and grade 1 elevations in ALT, AST, and ALP. This resolved after treatment with a 6-week oral prednisone taper; he resumed normal maintenance treatment with atezolizumab in Aug 2023 with no recurrence of transaminitis as of publication. This was the only irAE that this patient experienced since starting atezolizumab at diagnosis.

Today, the patient is alive and well (aged 63) and continues to experience minimal functional impairments due to his illness. He still works as a refrigerator mechanic with minor workplace accommodations. Throughout treatment, side effects have been minimal. His current ECOG at the time of this report is 0. He continues to receive atezolizumab maintenance therapy every 3 weeks and is monitored for progression via surveillance CT thorax/abdomen and neck scans every 3–4 months and clinical neurologic screening.

## 3. Discussion

In total, this patient has achieved 60 months (5 years) OS and 44 months (about 3.5 years) progression-free survival (PFS) and is still alive to date, drastically exceeding survival metrics for ES-SCLC. The median OS for ES-SCLC, even with the addition of ICIs, is approximately 12 months and the average 2-year survival rate is between 5.2 and 19.5% [3,8]. Additionally, this patient has maintained a high quality of life throughout treatment, developing few symptoms and maintaining functional capacity, which contrasts with the physical decline often experienced by ES-SCLC patients [9]. He has experienced only one irAE during treatment, a brief episode of transaminitis that resolved with prednisone taper. Overall, this case demonstrates that long-term survival can be accomplished with atezolizumab for ES-SCLC with preservation of functional capacity and minimal immune-related toxicity.

One of the most remarkable aspects of this patient’s case is the durability of response to atezolizumab. For various cancers, it has been reported that small subsets of patients achieve dramatic increases in long-term survival when treated with immunotherapy, even after discontinuation or interruptions [10]. This phenomenon has been observed in advanced melanoma, NSCLC, and renal cell cancer (RCC), which has led to an increase in 5-year survival rates and a plateau of Kaplan–Meier survival curves around 3 years from treatment initiation [11]. The most recent survival data from IMpower133 suggest that durable responses from immunotherapy may be having a similar effect on survival metrics for ES-SCLC. At 18 months follow-up, the treatment-arm of atezolizumab plus carboplatin and etoposide achieved 34% OS compared to the control-arm, which achieved 21% OS [12].

To our knowledge, this is the third published case of durable response to atezolizumab in ES-SCLC. Bernabe et al. (2024) reported a case of a patient who was part of the investigational arm in the IMpower133 study and similarly received atezolizumab as induction and maintenance therapy [13]. Maintenance atezolizumab was continued for approximately one year before it was discontinued due to toxicity, but this patient has achieved six years OS without any additional treatment [13]. Similarly, Konishi et al. (2023) reported a patient that achieved 35 months OS who received atezolizumab alongside carboplatin and etoposide for approximately 6 months before the development of disease progression, after which they were treated with palliative radiation and second-line chemotherapy [14]. Our case differs from these reports as our patient has remained on maintenance atezolizumab since diagnosis, without discontinuation due to toxicity or disease progression, and has seen a sustained survival benefit. Overall, these cases support the notion that it is possible to achieve durable responses to atezolizumab in a small subset of ES-SCLC patients.

Various research has been undertaken to uncover biomarkers that can predict a durable response to immunotherapy in ES-SCLC. Expression of PD-L1, the molecular target of atezolizumab, was not found to be predictive of survival or response rates in a retrospective analysis of the IMpower133 trial [15]. More promising results have been shown for biomarkers, such as transcriptional subtype SCLC-I, major histocompatibility complex (MHC) class I, and circulating tumor DNA (ctDNA); however, as of yet no biomarkers have been established for therapeutic decision making in ES-SCLC [16]. When accounting for our patient’s durable survival on atezolizumab, it is difficult to comment on the molecular mechanism underlying this response. In this case, we attempted enrollment into local studies for next-generation sequencing; however, the initial biopsy sample was insufficient, and the excellent response meant there were no available areas to re-biopsy. This represents a limitation of our study and showcases some barriers to obtaining this testing in practice. We also unfortunately did not have access to a liquid biopsy due to a lack of coverage for this testing in our single-payer publicly funded healthcare system, the lack of any clinical trials at our institution which would allow access to such testing, and the prohibitive cost to the patient. Nonetheless, this case ultimately highlights the need for further work to be conducted in this area so that patients who are most likely to respond to ICI therapy can be stratified appropriately.

It is also important to highlight some clinical characteristics that may have contributed to our patient’s long-term survival. Ma et al. (2022) performed a retrospective analysis showing that certain clinical characteristics were associated with greater OS in ES-SCLC, several of which our patient possesses, namely an ECOG of 0 at diagnosis, a positive response to first-line systemic therapy, the receipt of initiative thoracic irradiation therapy, and the absence of any brain, bone, or subcutaneous metastasis [17]. These characteristics may have contributed to their long-term survival independently in conjunction with the use of atezolizumab.

Furthermore, the role of thoracic radiation in our patient’s long-term survival should be highlighted. Radiation therapy has been hypothesized to have immune-modulating effects that can improve response to immunotherapy [18]. Specifically, it is thought to increase the expression of inflammatory mediators that attract T-cells to the tumor site and upregulate the expression of MHCs on the surface of tumor cells, which allows for synergistic benefits with the T-cell-enhancing effect of ICIs [18]. Currently, the most recent Canadian Consensus Guidelines have stated that all patients with a positive response to chemoimmunotherapy, good performance status, and limited metastasis can be considered for thoracic radiotherapy [19]. This is based on follow-up analysis of the CREST trial [19]. However, randomized control trial data showing the definitive efficacy of this approach are lacking. Notably, RAPTOR is an ongoing randomized phase 2/3 clinical trial that is investigating the impact on PFS and OS between patients who receive radiation therapy after 4–6 cycles of platinum-based chemotherapy and atezolizumab versus maintenance atezolizumab alone [20]. Our patient underwent virtually the same treatment regimen that is being tested in this trial and has experienced a durable and well-tolerated response. This is a promising finding and should provide an interesting opportunity for comparison once the results are published.

Finally, a unique aspect of our patient’s treatment regimen that may have also contributed to his durable responses was the decision to continue atezolizumab after disease progression. Currently, no randomized control trial data exist to support the use of immunotherapy post-disease progression. A retrospective study by Li et al. (2022) demonstrated the benefit of immunotherapy rechallenge (defined as treatment for greater than 6 weeks of immunotherapy post-progression) after disease progression in ES-SCLC on disease control rate, durable clinical benefit, and PFS, but no benefit in overall survival [21]. For our patient, atezolizumab was continued after radiologic evidence of mixed progression as discussed above, as part of a retrial of the initial chemoimmunotherapy induction regimen. He achieved almost complete resolution of his disease after this rechallenge and has shown no evidence of further progression while continuing maintenance atezolizumab. Thus, this case demonstrates that it may be appropriate to reconsider the need to discontinue maintenance immunotherapy in context with the degree and type of disease progression, initial response to induction therapy, and patient tolerability.

Inherent strengths of this case report include a detailed case description that includes clear rationale for all major treatment decisions made for this patient. Commentary and evidence are provided for three major treatment approaches in oncology: chemotherapy, immunotherapy, and radiation therapy. Weaknesses include the lack of generalizability of the findings, which is inherent to all case reports, and the lack of pathological data, which made it impossible to comment on the molecular mechanisms underlining this patient’s durable response.

## 4. Conclusions

In conclusion, this case demonstrates that it is possible for ES-SCLC patients to achieve well-tolerated, durable responses to atezolizumab that result in long-term survival and preservation of functional status. Our patient likely belongs to a small subset of ES-SCLC patients who are capable of experiencing sustained and durable responses to immunotherapy and his case highlights the need for further research into the clinical, genetic, and immunological factors governing this response.

## Data Availability

Due to the nature of the research, supporting data are not available.
